# Glycan-Glycan Interaction Determines *Shigella* Tropism toward Human T Lymphocytes

**DOI:** 10.1128/mBio.02309-17

**Published:** 2018-02-13

**Authors:** Ilia Belotserkovsky, Katja Brunner, Laurie Pinaud, Alexander Rouvinski, Mariano Dellarole, Bruno Baron, Gyanendra Dubey, Fatoumata Samassa, Claude Parsot, Philippe Sansonetti, Armelle Phalipon

**Affiliations:** a Molecular Microbial Pathogenesis Unit, Department of Cellular Biology of Infection, Institut Pasteur, Paris, France; b INSERM U1202, Paris, France; c Structural Virology Unit, Virology Department, Institut Pasteur, Paris, France; d CNRS UMR 3569, Paris, France; e Institut Pasteur, Molecular Biophysics Platform, Centre for Innovation and Technological Research, Paris, France; f CNRS UMR 3528, Paris, France; g Chaire de Microbiologie et Maladies Infectieuses, Collège de France, Paris, France; New York University School of Medicine

**Keywords:** GM1, LPS, T lymphocytes, type III secretion system (T3SS), actin, adaptive immunity, enteric bacteria, gangliosides, glycosylation, host-pathogen interactions, liposomes, outer membrane vesicles

## Abstract

Direct interactions between bacterial and host glycans have been recently reported to be involved in the binding of pathogenic bacteria to host cells. In the case of *Shigella*, the Gram-negative enteroinvasive bacterium responsible for acute rectocolitis, such interactions contribute to bacterial adherence to epithelial cells. However, the role of glycans in the tropism of *Shigella* for immune cells whose glycosylation pattern varies depending on their activation state is unknown. We previously reported that *Shigella* targets activated, but not nonactivated, human CD4^+^ T lymphocytes. Here, we show that nonactivated CD4^+^ T lymphocytes can be turned into *Shigella*-targetable cells upon loading of their plasma membrane with sialylated glycosphingolipids (also termed gangliosides). The *Shigella* targeting profile of ganglioside-loaded nonactivated T cells is similar to that of activated T cells, with a predominance of injection of effectors from the type III secretion system (T3SS) not resulting in cell invasion. We demonstrate that gangliosides interact with the O-antigen polysaccharide moiety of lipopolysaccharide (LPS), the major bacterial surface antigen, thus promoting *Shigella* binding to CD4^+^ T cells. This binding step is critical for the subsequent injection of T3SS effectors, a step which we univocally demonstrate to be dependent on actin polymerization. Altogether, these findings highlight the critical role of glycan-glycan interactions in *Shigella* pathogenesis.

## IMPORTANCE

Glycosylation of host cell surface varies with species and location in the body, thus contributing to species specificity and tropism of microorganisms. Cross talk by *Shigella*, the Gram-negative enteroinvasive bacterium responsible for bacillary dysentery, with its exclusively human host has been extensively studied. However, the molecular determinants of the step of binding to host cells are poorly defined. Taking advantage of the observation that human-activated CD4^+^ T lymphocytes, but not nonactivated cells, are targets of *Shigella*, we succeeded in rendering the refractory cells susceptible to targeting upon loading of their plasma membrane with sialylated glycosphingolipids (gangliosides) that are abundantly present on activated cells. We show that interactions between the sugar polar part of gangliosides and the polysaccharide moiety of *Shigella* lipopolysaccharide (LPS) promote bacterial binding, which results in the injection of effectors via the type III secretion system. Whereas LPS interaction with gangliosides was proposed long ago and recently extended to a large variety of glycans, our findings reveal that such glycan-glycan interactions are critical for *Shigella* pathogenesis by driving selective interactions with host cells, including immune cells.

## OBSERVATION

Mammalian cell surfaces are covered with a wide variety of glycans linked to proteins (glycoproteins and proteoglycans) and lipids (glycolipids). In addition to their multiple roles in cellular processes, these glycans also serve as target molecules for binding of pathogenic microorganisms and virulence factors, such as toxins (1). Such interactions contribute to the species specificity and tissue tropism of the pathogen; so far, this has been extensively studied mainly for viruses (2).

Molecular mechanisms of binding of *Shigella*, the Gram-negative enteroinvasive bacterium responsible for bacillary dysentery, to host cells are poorly understood. What has been mostly studied are the interactions of epithelial cells with the proteins of the type III secretion system (T3SS) and their role in cell invasion. The T3SS, a key element for *Shigella* pathogenicity, is a supramolecular syringe-like type III secretion apparatus (T3SA) enabling delivery of bacterial virulence effectors directly into the host cell cytoplasm (3). For example, the interaction between the hyaluronic acid receptor, the glycoprotein CD44, and the T3SS component IpaB appears to initiate the early steps of *Shigella* invasion (4). This molecular complex is anchored within specialized membrane microdomains enriched in cholesterol and sphingolipids that are critical to trigger contact-mediated activation of the T3SA (5). In addition, the interaction of some Ipa proteins, including IpaB, with α5β1 integrin may be an important factor in initiating the reorganization of the actin cytoskeleton necessary for bacterial internalization (6). The effectors OspE1, OspE2, and IcsA also have a role in bacterium-cell interaction by mediating adherence to the colonic epithelium following exposure to bile salts, which results in enhancement of cell invasion (7, 8). Interactions between the host cell membrane and the bacterial surface, independently of T3SS components, have been recently investigated, highlighting the importance of glycan-glycan interactions in mediating binding of *Shigella* to host epithelial cells (9).

Aiming at deciphering the mechanisms underlying the inefficient priming of host adaptive immunity upon *Shigella* infection, we studied the cross talk between the bacteria and T lymphocytes (10). We recently optimized a reporter tool to directly visualize T3SS effector injection by a fluorescence resonance energy transfer (FRET)-based β-lactamase assay, originally reported to monitor enteropathogenic *Escherichia coli* effector translocation (11). We found that besides invasion, the main *Shigella*-mediated targeting mechanism toward activated CD4^+^ T cells is “injection-only,” in which injection of T3SS effectors does not result in subsequent invasion (12). Interestingly, nonactivated CD4^+^ T cells, as opposed to activated ones, are neither invaded nor injected (12, 13). By taking advantage of the refractory state of nonactivated CD4^+^ T cells, we investigated the molecular mechanism underlying *Shigella* interaction with host immune cells. We succeeded in converting the nontargetable CD4^+^ T cells into targetable ones and demonstrated that polysaccharide-mediated bacterial binding to cell glycosphingolipids is essential for a selective targeting of human T lymphocytes by *Shigella*.

### Long-term, but not short-term, activated human primary CD4^+^ T lymphocytes are targeted by *Shigella*.

**FIG 1:**
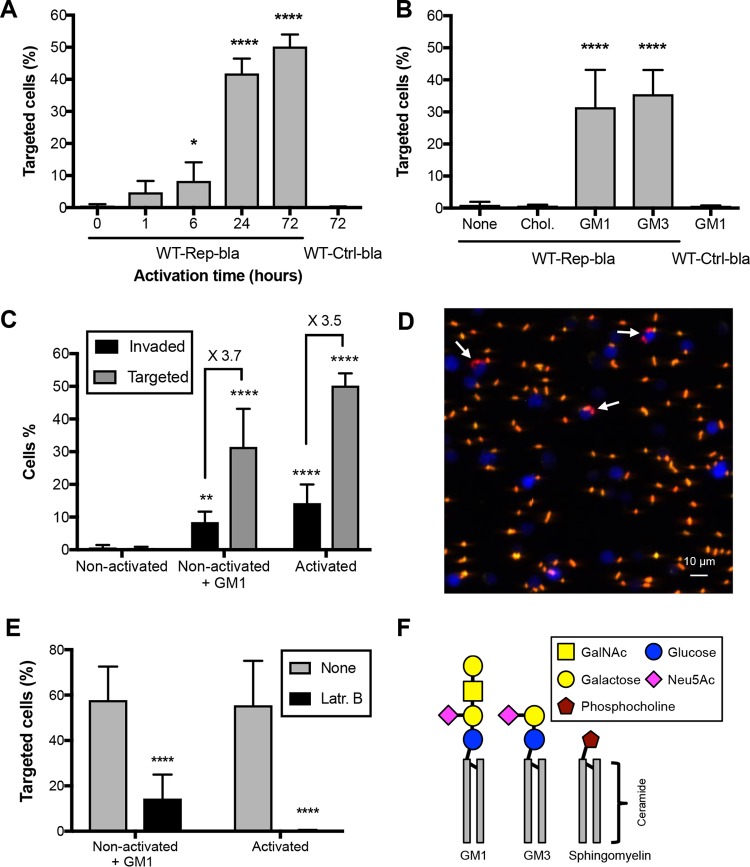
Plasma membrane ganglioside enrichment promotes *Shigella* targeting of otherwise refractory nonactivated CD4^+^ T lymphocytes. (A) Nonactivated CD4^+^ T cells isolated from human blood were activated using PMA for the indicated time periods. Cells were then infected with WT-Rep-bla (or WT-Ctrl-bla as a control), and the percentage of targeted cells was quantified by flow cytometry. (B) *Shigella* targeting of nonactivated CD4^+^ T cells loaded with GM1 or GM3 gangliosides or cholesterol (Chol.) was assessed as described for panel A. (C) Comparison of *Shigella* targeting-to-invasion ratios between nonactivated CD4^+^ T cells loaded or not loaded with GM1 and activated CD4^+^ T cells, upon infection with WT-GFP and WT-Rep-bla strains giving rise to GFP^+^ invaded cells and Blue^+^ targeted cells, respectively, assessed by flow cytometry. (D) Fluorescence microscopy of GM1-loaded nonactivated CD4^+^ T cells infected with WT-Rep-bla strain. Targeted cells are shown in blue. Extracellular bacteria stained by anti-LPS specific antibody are yellow-orange. Intracellular bacteria appear in red due to constitutive DsRed expression. Cells containing intracellular bacteria are marked with arrows. A representative merged image of maximal Z projection is shown. (E) Effect of actin polymerization inhibition by latrunculin B (Latr. B) on *Shigella* targeting of activated and GM1-loaded nonactivated CD4^+^ T cells, assessed as described for panel A. (F) Schematic representation of GM1 and GM3 ganglioside and sphingomyelin structure. (A, B, C, and E) Results represent the mean ± standard deviation from 3 independent experiments. One-way ANOVA was performed comparing all samples to nonactivated (A) or nonloaded (None) control (B). Two-way ANOVA was performed comparing all samples to the nonactivated (C) or nonloaded (None) (E) control group. *, *P* < 0.05; **, *P* < 0.005; ****, *P* < 0.00005. GalNAc, *N*-acetylgalactosamine; Neu5Ac, *N*-acetylneuraminic acid (sialic acid).

We previously showed that human primary CD4^+^ T lymphocytes activated for 3 days with phorbol 12-myristate 13-acetate (PMA), as opposed to nonactivated ones, are susceptible to *Shigella* invasion (13) and to injection-only T3SS effector (12). CD4^+^ T lymphocyte activation is a complex process including short-term (in the range of approximately minutes) and long-term (several hours to a few days) events taking place after initial stimulation (14). To investigate whether short-term activation might also result in promoting *Shigella* targeting of naive CD4^+^ T cells, *Shigella* infection was performed at different time points upon PMA activation using the WT-Rep-bla strain that we recently described, which allows monitoring of T3SS-mediated injection into host cells (12). The WT-Ctrl-bla strain not delivering the reporter T3SS effectors was used as a negative control (Fig. 1A). The percentage of targeted cells, corresponding to both injected-only and invaded cells, was quantified by flow cytometry. The percentage of targeted cells increased with time up to 40% at 24 h with no subsequent major increase up to 72 h postactivation (Fig. 1A). These data indicate that the susceptibility of human primary CD4^+^ T cells to *Shigella* targeting depends on cellular events requiring long-term activation.

### Nonactivated human primary CD4^+^ T lymphocytes become susceptible to *Shigella* targeting upon plasma membrane ganglioside enrichment.

Changes occurring in T cells during long-term activation include modification of the plasma membrane composition with the accumulation of cholesterol and major changes in the ganglioside content and, in particular, a dramatic increase in the GM1 content (15). To test whether artificially increased amounts of these lipids in the plasma membrane of nonactivated CD4^+^ T cells render these cells targetable by *Shigella*, we used the cell's ability to incorporate gangliosides and cyclodextrin-enclosed cholesterol into the plasma membrane upon brief incubation with these compounds. Cholesterol loading of nonactivated CD4^+^ T lymphocytes did not result in *Shigella* targeting (Fig. 1B), even when the amount of cell membrane-incorporated cholesterol almost reached that found in the plasma membrane of activated cells (see Fig. S1A in the supplemental material). In contrast, GM1 loading resulted in *Shigella* targeting (Fig. 1B) in a dose-dependent manner (Fig. S1B). The percentage of targeted cells was similar to that obtained for CD4^+^ T cells activated for 24 h (Fig. 1A). In addition, the percentage of targeted cells was higher than that of invaded cells, indicating that the GM1-loaded CD4^+^ T cells were mostly T3SS injected-only, similarly to the activated ones (Fig. 1C). Indeed, confocal microscopy revealed that most of the targeted cells did not contain intracellular bacteria (Fig. 1D and S1C), confirming injection-only as the major targeting mechanism toward human lymphocytes (12). To assess the contribution of the ganglioside carbohydrate moiety in the GM1-mediated *Shigella* targeting, we investigated if plasma membrane loading with GM3, a precursor of GM1 that possesses a smaller polar head with only one lactosyl residue decorated with sialic acid (Fig. 1F), induces *Shigella* targeting of nonactivated CD4^+^ T cells. Upon GM3 loading, nonactivated CD4^+^ T cells were rendered targetable, similarly to GM1-loaded cells (Fig. 1B). The GM3 dose-response curve was similar to that of GM1 (Fig. S1B). These findings indicate that sialyl lactose coupled to ceramide is sufficient to trigger *Shigella* T cell targeting, while the additional *N*-acetyl lactose and galactose residues do not contribute to this phenomenon. Notably, *Shigella* targeting was significantly reduced in both activated CD4^+^ T cells and GM1-loaded nonactivated CD4^+^ T cells, compared to their nontreated counterparts, following incubation with latrunculin B, an inhibitor of actin polymerization (Fig. 1E). This formally demonstrates the requirement of actin for T3SS effector delivery into human CD4^+^ T lymphocytes, as previously reported for epithelial cells (16).

10.1128/mBio.02309-17.2 FIG S1  Plasma membrane ganglioside enrichment promotes *Shigella* targeting of otherwise refractory nonactivated CD4^+^ T lymphocytes. Download FIG S1, PDF file, 9.8 MB. Copyright © 2018 Belotserkovsky et al. 2018 Belotserkovsky et al. This content is distributed under the terms of the Creative Commons Attribution 4.0 International license.

### T lymphocyte plasma membrane gangliosides promote bacterial binding by interacting with the O-antigen (O-Ag) polysaccharide moiety of *Shigella* lipopolysaccharide (LPS).

**FIG 2:**
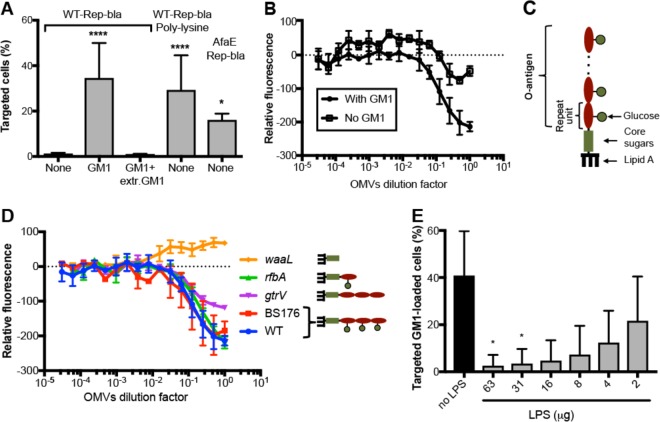
LPS O-Ag of *Shigella* interacts with GM1. (A) Nonactivated CD4^+^ T cells were loaded with GM1, washed, and then incubated or not incubated with GM1 (+ extr. GM1, 64 μM) before WT-Rep-bla infection. Alternatively, poly-l-lysine-coated or AfaE-expressing WT-Rep-bla *Shigella* was used to infect nonactivated CD4^+^ T cells. Cell targeting was assessed by flow cytometry. (B) Measurement of fluorescence upon incubation of fluorescent liposomes containing or not containing GM1 with a dilution series of *Shigella* OMVs. (C) Schematic representation of *Shigella* LPS structure. (D) Fluorescence measurements as described for panel B using OMVs from *Shigella* mutant strains. (B and D) Mean ± standard deviation from 3 independent experiments performed with at least two different preparations of OMVs for each tested strain. (E) LPS purified from the WT *S. flexneri* 5a strain (Table S1) was added at the indicated concentrations to GM1-loaded, nonactivated CD4^+^ T cells prior to infection with WT-Rep-bla. Bacterial targeting was assessed as described for panel A. (A and E) Means ± standard deviations from 3 independent experiments are shown. One-way ANOVA was performed comparing all samples to the control group (None or no LPS, respectively). *, *P* < 0.05; ****, *P* < 0.00005.

Glycan-glycan interactions have been recently reported to promote high-affinity interactions of pathogenic bacteria, including *Shigella*, with host cells (9). To investigate whether T lymphocyte plasma membrane gangliosides contribute to *Shigella* T3SS-mediated targeting by promoting bacterial binding, *Shigella* infection of nonactivated CD4^+^ T cells loaded with GM1 was performed in the presence or in the absence of GM1 in the cell culture medium. As shown in Fig. 2A, *Shigella* targeting was prevented in the presence of extracellular GM1, in a dose-dependent manner (Fig. S2A), suggesting saturation of binding sites on bacterial surface by GM1. To further assess the importance of the binding step in *Shigella* T cell targeting, binding of bacteria to nonactivated cells was forced by either coating of WT-Rep-bla bacteria with positively charged poly-l-lysine (which promotes nonspecific electrostatic interaction with the negatively charged host cell membrane) or using WT-Rep-bla expressing the *E. coli* adhesin AfaE (which binds the host cell surface protein CD55) (17). In both cases, efficient targeting of nonactivated T lymphocytes was observed (Fig. 2A), indicating that promoting binding of bacteria to nonactivated T cells is sufficient to render these cells targetable by *Shigella*.

10.1128/mBio.02309-17.3 FIG S2  LPS O antigen of *Shigella* interacts with GM1 or GM3. Download FIG S2, PDF file, 2 MB. Copyright © 2018 Belotserkovsky et al. 2018 Belotserkovsky et al. This content is distributed under the terms of the Creative Commons Attribution 4.0 International license.

10.1128/mBio.02309-17.4 TABLE S1  List of the strains used in this study. Download TABLE S1, PDF file, 0.1 MB. Copyright © 2018 Belotserkovsky et al. 2018 Belotserkovsky et al. This content is distributed under the terms of the Creative Commons Attribution 4.0 International license.

To demonstrate the binding of *Shigella* to GM1, we used a cell-free system mimicking both bacterial and cell membranes. Fluorescent liposomes whose composition includes cholesterol and sphingomyelin to mimic membrane domains, which are the natural environment of GM1 in the plasma membrane, were produced with or without GM1. *Shigella* outer membrane vesicles (OMVs) that are spontaneously secreted by Gram-negative bacteria and contain all the bacterial surface molecules were prepared (18). We first established that incubation of cholera toxin subunit B (CTxB), which specifically binds to GM1, with fluorescent GM1-containing, but not GM1-devoid, liposomes resulted in fluorescence quenching (Fig. S2B). Thus, fluorescent liposomes containing or not containing GM1 were then mixed with increasing concentrations of OMVs, and the resulting fluorescence was measured. As opposed to fluorescent GM1-devoid liposomes, the fluorescence of the GM1-containing liposomes was quenched proportionally to the OMV concentration (Fig. 2B), indicating that GM1 interacts with *Shigella* OMVs. Sphingomyelin, which is one component of both GM1-containing and GM1-devoid liposomes, differs from GM1 only by the surface-exposed polar head (Fig. 1F). Therefore, the absence of OMVs binding to GM1-devoid liposomes (no fluorescence quenching), as opposed to binding to GM1-containing liposomes (fluorescence quenching), indicates that binding does not involve the ceramide part but the sugar moiety of GM1.

In a recent study using glycan microarrays, numerous host glycan structures have been shown to bind to lipopolysaccharide (LPS) or lipooligosaccharide of several bacterial pathogens (9). LPS is the most abundant molecule on the surface of *Shigella,* extending for about 35 nm from the outer membrane (19). We thus tested whether LPS was the bacterial partner of GM1. *Shigella* LPS consists of a lipid A part inserted into the bacterial outer membrane and a polysaccharide chain made of a core region and the O-Ag, composed of a repeating unit whose composition varies between *Shigella* species and serotypes (20). For *Shigella flexneri* serotype 5a, the repeating unit comprises three rhamnose and one *N*-acetyl glucosamine residue, with a glucose residue specifically branched on the central rhamnose (Fig. 2C). The two predominant O-Ag lengths on the bacterial surface consist of ~15 and ~100 repeating units (RU). OMVs were purified from three *Shigella* strains impaired in LPS biosynthesis, the *gtrV* mutant displaying O-Ag repeating units deprived of the glucose residue, the *rfbA* mutant expressing an O-Ag made of ~15 repeating units only, and the *waaL* mutant lacking an O-Ag (19), and from a virulence plasmid-cured mutant—BS176—not expressing virulence factors (including T3SS) but exhibiting similar LPS as the wild-type (WT) strain. As shown in Fig. 2D, fluorescence quenching of GM1-containing liposomes occurred upon incubation with OMVs from the *gtrV* and the *rfbA* mutant, indicating that binding was independent of the repeating unit (RU) composition and the chain length of O-Ag, respectively. In contrast, fluorescence quenching of GM1-containing liposomes did not occur upon incubation with OMVs from the *waaL* mutant, showing that binding was dependent on the presence of the O-Ag. It is noticeable that a similar morphology was observed for OMVs from the *waaL* mutant and the WT strain (Fig. S2C). These results indicate that none of the proteins encoded by the virulence plasmid are involved in binding of bacteria to GM1 and strongly suggest that the LPS O-Ag interacts with the sugar moiety of GM1 to promote bacterial binding to T cells. To further document O-Ag–GM1 interaction, GM1-loaded nonactivated CD4^+^ T cells were infected with *Shigella* in the presence or absence in the cell culture medium of LPS purified from the corresponding bacterial strain, and the percentage of targeted cells was measured as previously described. As shown in Fig. 2E, the percentage of targeted cells was decreased in the presence of LPS, in a dose-dependent manner. Similar results were obtained with GM3-loaded nonactivated CD4^+^ T cells (Fig. S2D). Altogether, these findings show that glycan-glycan interactions promote bacterial binding to host cells, thus driving *Shigella* targeting of T lymphocytes.

### Conclusion.

Our study brings new insights into how pathogens use glycans to target host cells, especially when they are devoid of particular weapons such as adhesins to trigger strong cell adhesion or bind to a particular host cell receptor. The novelty of our findings resides in the fact that interactions between *Shigella* O-Ag and host cell glycans, reported to promote bacterial binding to epithelial cells (9), are shown here to dictate the *Shigella* tropism toward cells of the adaptive immunity. Previous studies aiming at understanding bacterial-host cell interaction suggested a role for LPS and cellular glycans, including gangliosides, by using different cell types and various *Shigella* strains. Bacterial strains defective in LPS O-Ag expression were shown to be impaired in their ability to attach to and subsequently invade intestinal epithelial cells *in vitro* (21), and the invasion rate was reduced in the presence of LPS as well as some sugars in the cell culture medium (22, 23). *Shigella* LPS was shown to bind a large array of immobilized glycans commonly present on the human cell surface, but those corresponding to the sugar moiety of GM1 and GM3 were not present on the array (9). Gangliosides were reported to inhibit specific binding of LPS to its receptor on human monocytes. Interactions were shown to take place between the sugar polar part of the gangliosides and the polysaccharide moiety of LPS (24). In the current study, we used a cell-free experimental model mimicking the native environment of both partners, i.e., bacterial OMVs naturally released by the bacteria and liposome-embedded gangliosides, to avoid the likely loss of native conformation and spatial orientation of both gangliosides and LPS resulting from techniques used in previous studies. In addition, a vesicle-based system permits dynamic lateral diffusion of the surface molecules, enabling multivalent interactions between the vesicles, thus mimicking the host-bacterium interface.

Our findings, along with others previously showing the contribution of glycoproteins (the hyaluronic acid receptor CD44 and the α5β1 integrin) to *Shigella* binding to epithelial cells (4, 6), suggest that *Shigella* might bind a spectrum of glycans present on protein and/or lipid clusters in the host plasma membrane domain. We hypothesize that the multivalent interactions occurring between *Shigella* LPS O-Ag and cell surface glycans promote remodeling of the cell membrane, resulting in the formation of larger membrane domains that are known to promote T3SS activation (5). The fact that injection of T3SS effectors into T lymphocytes is shown in this study to be dependent on actin polymerization further supports the above hypothesis, since actin filaments have been shown to contribute to the stabilization of these larger membrane domains (25).

Previous data showed that *Shigella dysenteriae* 1 preferentially binds highly glycosylated mucins isolated from human colon compared to guinea pig (less binding), and rat (no binding at all) (26), supporting the notion that *Shigella*’s strict tropism toward humans might be dictated by its ability to bind human-specific types of glycans. Beyond the potential impact in understanding host specificity, our results suggest that the outcome of *Shigella* binding to a wide spectrum of cell surface glycans is likely to drive selective targeting not only of different T lymphocyte subsets but also of other immune cells, for which glycosylation matters for their functions (27), therefore extending the importance of such interactions to *Shigella* pathogenesis.

### Bacterial strains and growth media.

Bacterial strains used in the study are listed in Table S1. Bacteria were grown at 37°C on Trypticase soy (TCS) (Becton, Dickinson) agar plates containing 0.01% Congo red (Serva), supplemented as needed with 10 μg/ml chloramphenicol or 50 μg/ml kanamycin. A Congo red-positive colony was picked for an overnight (O/N) culture at 37°C in TCS medium with appropriate antibiotics, followed by a subculture in an antibiotic-free medium to grow the bacteria to mid-logarithmic phase (optical density at 600 nm [OD_600_] of 0.8 to 1).

### Isolation and activation of primary CD4^+^ T cells from human blood.

Samples were obtained from the Etablissement Français du Sang (EFS). This study has been approved by the Institut Pasteur’s ethical and medical committee under the agreement number HS2015-24009. Peripheral blood mononuclear cells (PBMCs) were purified by density separation on Ficoll-Paque Plus (GE Healthcare) upon centrifugation at room temperature (RT) for 30 min at 800 × *g* without a pause. Nonactivated CD4^+^ T lymphocytes were isolated using Miltenyi MicroBeads naive CD4^+^ T cell kit II according to the manufacturer’s instructions. Cells were cultured in RPMI 1640 medium supplemented with 10% heat-inactivated fetal calf serum (FCS), 100 U/ml penicillin, and 100 μg/ml streptomycin (Gibco) at 37°C in a 5% CO_2_ incubator. The purity was verified on a FACSCanto II flow cytometer (BD Bioscience) using anti-human CD45RA and CD4 antibodies according to manufacturer’s instructions (purity was typically above 85%). Activation of lymphocytes was performed in the culture medium supplemented with 5 ng/ml phorbol 12-myristate 13-acetate (PMA; Sigma P1585) and 100 ng/ml recombinant human interleukin-2 (IL-2) (PeproTech).

### *Shigella* injection and invasion into cells.

Cells were washed in RPMI medium and seeded in round-bottom 96-well plates at 5 × 10^5^ cells per well in 100 μl in the same medium. When indicated, the medium was supplemented with 64 μM GM1 (Sigma-Aldrich) or GM3 (Matreya) gangliosides or 46 μg/ml water-soluble cholesterol (Sigma-Aldrich) for 1 h at 37°C, and cells were washed again in RPMI medium. Higher concentrations of water-soluble cholesterol and gangliosides were toxic for the nonactivated CD4^+^ T cells.

For competitive inhibition analysis, a dilution series of GM1, GM3, or LPS (*Shigella flexneri* M90T 5a, purified in-house) was added to the culture medium of GM1- or GM3-loaded T cells and allowed to equilibrate for 10 min prior to bacterial coculture.

F-actin inhibition was achieved by 1 μM latrunculin B (Sigma-Aldrich) treatment 20 min before the infection. For poly-l-lysine coating, bacteria were washed in phosphate-buffered saline (PBS; pH 7.4), incubated with 10 μg/ml of poly-l-lysine (Sigma-Aldrich), and washed twice with the same buffer. For cell infections, bacteria were adjusted to an OD_600_ of 0.2, and 50 μl of this suspension was centrifuged onto the cells at 300 × *g* for 5 min to achieve a multiplicity of infection (MOI) of 10. Infection was allowed to proceed for 1 h at 37°C in a 5% CO_2_ incubator. To assess cell invasion, *Shigella* constitutively expressing green fluorescent protein (WT-GFP) was used (13). Briefly, 50 μg/ml of gentamicin (Sigma-Aldrich) was used to kill extracellular bacteria after 1 h of infection, and the cells were incubated for 3 additional hours at 37°C before acquisition with a flow cytometer. Invaded cells were identified as a GFP^high^ population among live (4′,6-diamidino-2-phenylindole-negative [DAPI^−^]) cells. To assess T3SS injection into host cells, the WT-Rep-bla strain was used (12). Briefly, these bacteria express a plasmid coding for the first 80 amino acids of the OspD1 T3SS effector translationally fused to the TEM3-M182T variant of the β-lactamase enzyme. Upon 1 h of infection with this strain, infected cells were supplemented with 2.5 mM water-soluble probenecid (Biotium), 1 μM CCF2-AM (Thermo Fisher Scientific), and 50 μg/ml of gentamicin. After an additional hour of incubation at 37°C, cells containing cleaved CCF2 (Blue^pos^) were detected with a FACSCanto II flow cytometer (BD Bioscience) using a 405-nm laser as recently described (12). For microscopy examination, cells were transferred onto glass coverslips precoated with 10 μg/ml poly-l-lysine, centrifuged for 1 min at 18 × *g*, fixed with 4% paraformaldehyde for 15 min at RT, and washed in PBS. For labeling of extracellular bacteria, cells were incubated with anti-*Shigella* LPS antibodies (laboratory collection) at a 1/300 dilution in PBS supplemented with 0.1% bovine serum albumin (BSA) for 1 h, followed by washing and subsequent incubation with Cy5-conjugated anti-rabbit Ig antibody diluted at a 1/500 dilution in the same buffer. Intracellular bacteria were detected using their intrinsic fluorescence due to DsRed protein expression. Images were acquired on a spinning-disk confocal microscope (Zeiss Axiovert 200 equipped with a confocal spinning-disk head [Yokogawa CSU22; PerkinElmer]) and visualized using Fiji software (https://imagej.net/Fiji/Downloads).

### OMV isolation and quantification.

OMVs were isolated from *Shigella* culture based on the protocol adapted from reference 18. Briefly, bacteria were grown to mid-log phase in 400 ml TCS, and the bacterial cells were removed from suspension by centrifugation at 5,000 × *g* for 20 min at 4°C. The supernatant was concentrated by filtering through 100-kDa filters using a stirred ultrafiltration cell (Millipore) until the volume was reduced to about 5 ml. Then, the solution was filtered through 0.22-μm-pore-size Minisart syringe filters (Sartorius) to remove residual bacteria, and OMVs were collected by centrifugation at 100,000 × *g* for 1 h at 4°C. The pellet was resuspended in 100 μl PBS. To determine the concentration of OMVs, a protocol modified from the work of Pospichalova et al. for flow cytometry analysis of fluorescently labeled microvesicles was used (28). Briefly, a small sample of OMVs was diluted 100 times in PBS and stained with 5 μg/ml CellMask deep red plasma membrane stain (Thermo Fisher) for 10 min at RT. Then, 50 μl of 0.45-μm Sphero nano-fluorescent yellow beads (Spherotech) was added, and the mix was analyzed using a FACSCanto II flow cytometer (BD Bioscience) using 488- and 633-nm lasers. The OMV concentration was calculated based on the following formula: OMV concentration = (OMVs counted/beads counted) × bead concentration.

### Liposome preparation.

Fluorescent large unilamellar liposomes (LUVs) were prepared by the film-hydration method (29). Briefly, 1,2-dioleoyl-*sn*-glycero-3-phosphocholine (DOPC), sphingomyelin (SM), cholesterol, and 2-dioleoyl-*sn*-glycero-3-phosphoethanolamine-*N*-(7-nitro-2-1,3-benzoxadiazol-4-yl) (NBD-DPPE) at a molar ratio of 2:1:2:0.06, respectively, or DOPC, SM, GM1, cholesterol, and NBD-DPPE at a molar ratio of 2:1:1:2:0.06, respectively, were mixed in a glass tube (9 mM total lipid). The suspension was dried with a SpeedVac concentrator (Thermo Electron; RVT400) for at least 1 h at RT. The thin lipid film was rehydrated in PBS by vortexing in the presence of 180-μm acid-washed glass beads (Sigma). The multilamellar vesicles formed during the rehydration were subjected to 10 freeze-thaw cycles using liquid N_2_ and a heat block (Thermomixer Comfort; Eppendorf). To form 200-nm-diameter-sized LUVs, the multilamellar vesicles were extruded 21 times through a polycarbonate filter with a pore size of 200 nm. Except for GM1 (Sigma), all the lipids as well as the extrusion system were from Avanti Polar Lipids.

### OMV-liposome interaction.

The concentration of OMVs isolated from different mutants was adjusted with PBS to that of OMVs isolated from the WT *Shigella* strain. Then, OMVs were serially diluted by 2-fold in PBS (10-μl final volume), mixed with 10 μl of 225 μM fluorescent NBD-DPPE-containing liposomes, and incubated for 15 min at RT. The fluorescence was detected using a Monolith NT.115 instrument (Nanotemper) according to the manufacturer’s guidelines. The data were analyzed using the MO. Affinity analysis software based on initial fluorescence readout while normalizing between the different experiments using fluorescence of liposomes alone.

### Data presentation and statistical analysis.

Prism 6.0 (GraphPad Software, Inc.) was used for graphs and statistical analyses. Means and standard deviations are represented. One-way and two-way statistical analyses of variance (ANOVAs) were performed for experiments with one and two groups of data, respectively. Significant statistical differences are indicated by asterisks in the figures. The Illustrator CS5 software (Adobe) was used to assemble figures.

10.1128/mBio.02309-17.1 TEXT S1  Supplemental methods. Download TEXT S1, PDF file, 0.1 MB. Copyright © 2018 Belotserkovsky et al. 2018 Belotserkovsky et al. This content is distributed under the terms of the Creative Commons Attribution 4.0 International license.
