# Supplementary material for: Glycan-Glycan Interaction Determines Shigella Tropism toward Human T Lymphocytes
Source: mBio. 2018 Feb 13;9(1):e02309-17. doi: 10.1128/mBio.02309-17 (PMC5821077; doi:10.1128/mBio.02309-17)
Supplement: FIG S1 [file mbo001183724sf1.pdf]

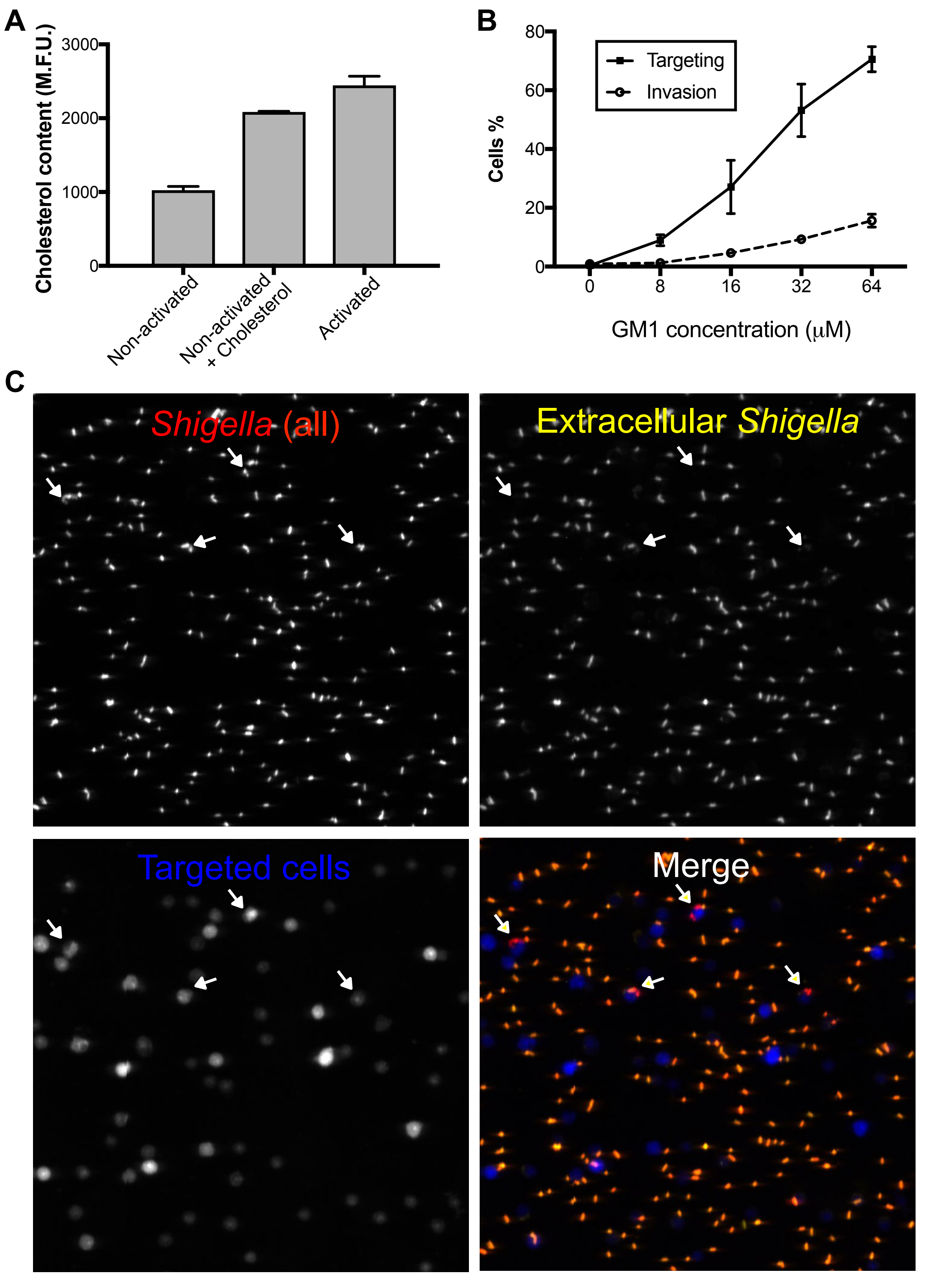

FIG S1. A) Cholesterol content of activated and non-activated CD4^{+} T cells loaded or not with water-soluble cholesterol assessed by flow cytometry upon Filipin staining. Representative results of 3 independent experiments. **B)** GM1 dose-dependent effect on *Shigella* invasion and targeting of non-activated CD4^{+} T cells assessed by flow cytometry as in Fig. 1C. Mean ± SD of 3 independent experiments is shown. **C)** Fluorescence microscopy of GM1-loaded non-activated CD4^{+} T cells infected with

WT-Rep-bla *Shigella* similar to Fig. 1 but with the presentation of larger view fields and individual fluorescent channels in gray-scale (top and left down panels). In the merged panel (right down), targeted cells appear in blue, extracellular bacteria detected by specific anti-LPS antibody in yellow-orange, and intracellular bacteria in red due to constitutive DsRed expression. The location of intracellular bacteria is marked with arrows in all panels. A representative image of maximal Z projection made using confocal fluorescence microscope is shown. M.F.U. – Mean Fluorescence Unit.
