# Supplementary material for: Glycan-Glycan Interaction Determines Shigella Tropism toward Human T Lymphocytes
Source: mBio. 2018 Feb 13;9(1):e02309-17. doi: 10.1128/mBio.02309-17 (PMC5821077; doi:10.1128/mBio.02309-17)
Supplement: FIG S2 [file mbo001183724sf2.pdf]

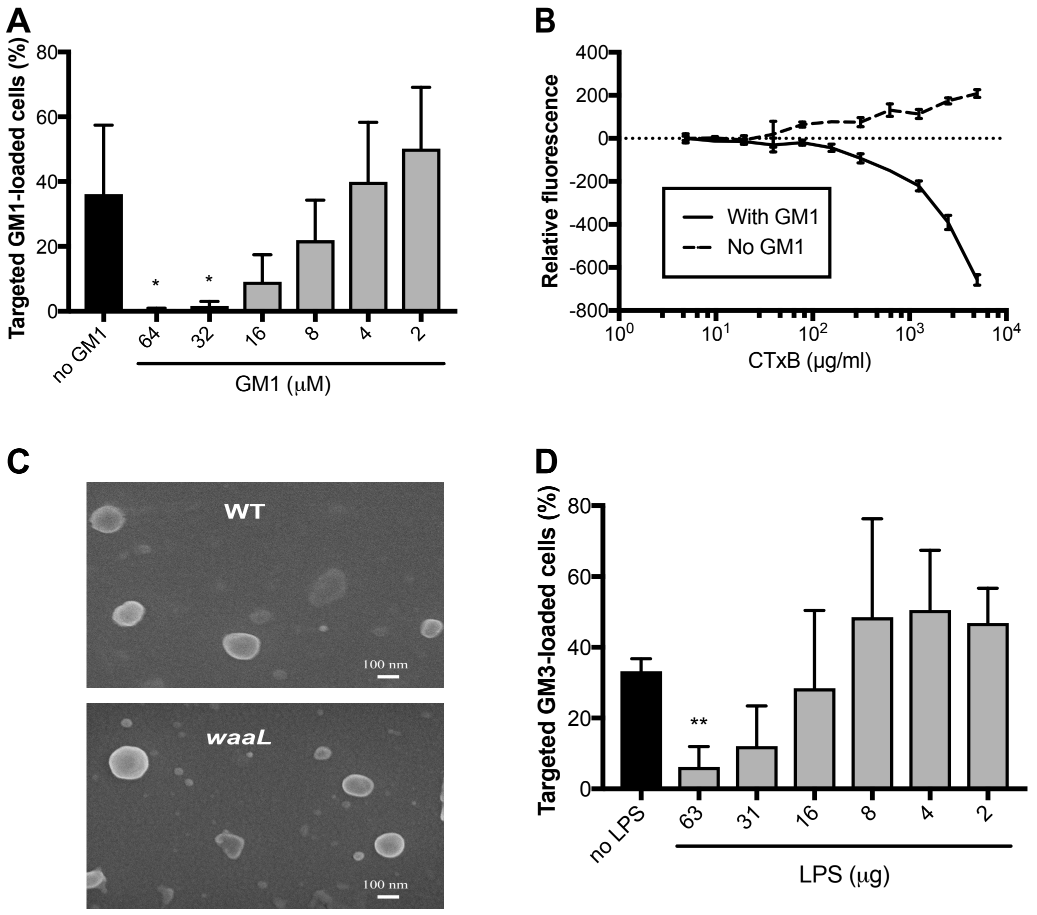

FIG S2. A) GM1-loaded, non-activated CD4$^+$ T cells were incubated with a dilution series of GM1 concentration prior to infection with WT-Rep-bla. Cell targeting was assessed by flow cytometry as described in Fig.2A and E. **B)** Measurement of fluorescence upon incubation of fluorescent liposomes containing or not GM1 with serially diluted cholera toxin B subunit (CTxB). Mean ± SD of 3 independent experiments. **C)** Scanning electron microscopy images of OMVs isolated from the supernatant of WT (top) and *waaL* (bottom) *Shigella* strains. **D)** LPS purified from WT *S. flexneri* 5a strain (Table S1) was added at the indicated concentrations to GM3-loaded, non-activated CD4$^+$ T cells prior to infection with WT-Rep-bla. Bacterial targeting was assessed by flow cytometry. (**A** and **D**) Mean ± SD of 3 or 2 independent experiments, respectively. One-way Anova analysis was performed

comparing all samples to the control group (no GM1 or no LPS, respectively). *$P<0.05$, **$P<0.01$.
