# Supplementary material for: Glycan-Glycan Interaction Determines Shigella Tropism toward Human T Lymphocytes
Source: mBio. 2018 Feb 13;9(1):e02309-17. doi: 10.1128/mBio.02309-17 (PMC5821077; doi:10.1128/mBio.02309-17)
Supplement: TABLE S1 [file mbo001183724st1.pdf]

Table S1. List of the strains used in the study

Strain name	Description	Reference
WT	*Shigella flexneri* 5a wild- type strain M90T	(1)
WT-GFP	M90T constitutively expressing green fluorescent protein	(2)
WT-Rep-bla	M90T reporter strain constitutively expressing DsRed fluorescent protein and the first 80 amino acids of the OspD1 T3SS effector translationally fused to TEM3-M182T β-lactamase	(3)
WT-Ctrl-bla	Similar to Rep-bla but possessing a deletion of the first 30 amino acids of the OspD1 coding sequence, therefore not secreted	(3)
AfaE-Rep-bla	Rep-bla transformed with pIL22 plasmid (4) that encodes for AfaE adhesin of *E. coli*	This study
BS176	Virulence plasmid-cured derivative of WT	(5)
rfbA	A M90T mutant possessing a short version of LPS O-antigen	(6)
gtrV	A M90T mutant possessing LPS O-antigen devoid of glucose	(6)
waaL	A M90T mutant devoid of the LPS O-antigen	(6)
