# Supplementary material for: Glycan-Glycan Interaction Determines Shigella Tropism toward Human T Lymphocytes
Source: mBio. 2018 Feb 13;9(1):e02309-17. doi: 10.1128/mBio.02309-17 (PMC5821077; doi:10.1128/mBio.02309-17)
Supplement: TEXT S1 [file mbo001183724s1.pdf]

Supplementary Methods

Assessment of cellular cholesterol content

Cells were incubated with 100 ug/mL Filipin (Sigma-Aldrich) for 15 min at RT. The relative fluorescence of the cells was detected by standard flow cytometry using a FACS Canto II flow cytometer (BD Bioscience) and FlowJo v.10.0.8 software for data analysis.

Scanning electron microscopy (SEM) of OMVs

Five μL of original OMVs preparation were spotted onto the electron microscopy grids (FCF300-Cu; EMS, USA), fixed in 2% glutaraldehyde and 2% paraformaldehyde in 0.1 M sodium cacodylate buffer (0.1 M, pH 7.2) for 30 min at 25°C. Grids were washed twice with cacodylate buffer, post-fixed using 1% osmium tetroxide for 30 min at 25°C, washed twice with cacodylate buffer and dehydrated through successive washes using ethanol solutions of 25%, 50%, 75%, 95%, 100% – two 10 min washes in each solution. Subsequently, grids were critical-point dried (CPD300; Leica, Germany), coated with gold-palladium (~8 nm) using ion beam coater (Gatan, USA) and observed by JSM 6700 scanning electron microscopy (Jeol, Japan).

References

1. **Sansonetti PJ**, **Kopecko DJ**, **Formal SB**. 1982. Involvement of a plasmid in the invasive ability of *Shigella flexneri*. Infection and Immunity **35**:852–860.

2. **Jaumouille V**, **Francetic O**, **Sansonetti PJ**, **Tran van Nhieu G**. 2008. Cytoplasmic targeting of IpaC to the bacterial pole directs polar type III secretion in *Shigella*. EMBO J **27**:447–457.

3. **Laurie P**, **Fatoumata S**, **Ziv P**, **Mariana F**, **Ilia B**, **Claude P**, **Philippe S**, **François-Xavier C-V**, **Armelle P**. Injection of T3SS effectors not resulting in invasion is the main targeting mechanism of *Shigella* towards human lymphocytes. Proc Natl Acad Sci U S A, in press. (Control number 2017-07098).

4. **Clerc P**, **Sansonetti PJ**. 1987. Entry of *Shigella flexneri* into HeLa cells: evidence for directed phagocytosis involving actin polymerization and myosin accumulation. Infection and Immunity **55**:2681–2688.

5. **Sansonetti PJ**, **Mounier J**. 1987. Metabolic events mediating early killing of host cells infected by *Shigella flexneri*. Microb Pathog **3**:53–61.

6. **West NP**, **Sansonetti P**, **Mounier J**, **Exley RM**, **Parsot C**, **Guadagnini S**, **Prevost M-C**, **Prochnicka-Chalufour A**, **Delepierre M**, **Tanguy M**, **Tang CM**. 2005. Optimization of virulence functions through glucosylation of *Shigella* LPS. Science **307**:1313–1317.
